# *Schistosoma mansoni* Soluble Egg Antigens Induce Expression of the Negative Regulators SOCS1 and SHP1 in Human Dendritic Cells via Interaction with the Mannose Receptor

**DOI:** 10.1371/journal.pone.0124089

**Published:** 2015-04-21

**Authors:** Elsenoor J. Klaver, Loes M. Kuijk, Thisbe K. Lindhorst, Richard D. Cummings, Irma van Die

**Affiliations:** 1 Department of Molecular Cell Biology and Immunology, VU University Medical Center, Amsterdam, The Netherlands; 2 Otto Diels Institute of Organic Chemistry, Christiana Albertina University of Kiel, Kiel, Germany; 3 Department of Biochemistry, Emory University School of Medicine, Atlanta, Georgia, United States of America; Queensland Institute of Medical Research, AUSTRALIA

## Abstract

Schistosomiasis is a common debilitating human parasitic disease in (sub)tropical areas, however, schistosome infections can also protect against a variety of inflammatory diseases. This has raised broad interest in the mechanisms by which *Schistosoma* modulate the immune system into an anti-inflammatory and regulatory state. Human dendritic cells (DCs) show many phenotypic changes upon contact with *Schistosoma mansoni* soluble egg antigens (SEA). We here show that oxidation of SEA glycans, but not heat-denaturation, abrogates the capacity of SEA to suppress both LPS-induced cytokine secretion and DC proliferation, indicating an important role of SEA glycans in these processes. Remarkably, interaction of SEA glycans with DCs results in a strongly increased expression of Suppressor Of Cytokine Signalling1 (SOCS1) and SH2-containing protein tyrosine Phosphatase-1 (SHP1), important negative regulators of TLR4 signalling. In addition, SEA induces the secretion of transforming growth factor β (TGF-β), and the surface expression of the costimulatory molecules Programmed Death Ligand-1 (PD-L1) and OX40 ligand (OX40L), which are known phenotypic markers for the capacity of DCs to polarize naïve T cells into Th2/Treg cell subsets. Inhibition of mannose receptor (MR)-mediated internalization of SEA into DCs by blocking with allyl α-D-mannoside or anti-MR antibodies, significantly reduced SOCS1 and SHP1 expression. In conclusion, we demonstrate that SEA glycans are essential for induction of enhanced SOCS1 and SHP1 levels in DCs via the MR. Our data provide novel mechanistic evidence for the potential of *S*. *mansoni* SEA glycans to modulate human DCs, which may contribute to the capacity of SEA to down-regulate inflammatory responses.

## Introduction

Parasitic helminths (worms) have evolved to suppress inflammatory responses of the immune system to survive in their hosts [1]. Interestingly, these properties have equipped them with the capacity to reduce the severity of inflammatory diseases within that host [1,2]. Evidence from epidemiological studies indicates an inverse relationship between the incidence of helminth infections and the occurrence of immune-related diseases including allergies, multiple sclerosis (MS) and inflammatory bowel disease (IBD) [3,4,5]. In experimental autoimmune encephalomyelitis, a mouse model for MS, eggs of *Schistosoma mansoni*, a parasitic trematode bloodfluke, reduced disease severity in established disease, while pre-established infection with live *S*. *mansoni* delayed onset and significantly reduced incidence of the disease [6]. In TNBS and DSS colitis models, two mouse models for IBD, *S*. *mansoni* was shown to ameliorate colitis [7,8] and infection with *S*. *mansoni* has beneficial effects in collagen-induced arthritis [9].

The effect of helminths to reduce the severity of inflammation within their host has been attributed to their capacity to modulate the host’s immune response. A key cell type in this pathway is the dendritic cell (DC), which is an antigen-presenting cell that plays a central role within the immune system in determining the type of effector T cell response via the recognition of pathogen-derived or self-antigens. DCs are well equipped to govern the development of naïve T helper (Th) cells towards Th1, Th2, or regulatory T cell (Treg) phenotypes, depending on the information that is received from sampled antigens. LPS-activated DCs polarize naïve T cells towards a Th1 response, by production of pro-inflammatory cytokines such as interleukin 12 (IL-12). On the other hand, infection with helminths like *S*. *mansoni* leads to Th2 and Treg responses, driven by DCs among other cells, via the increase in IL-10 and/or transforming growth factor β (TGF-β) levels, and surface expression of specific costimulatory molecules like Programmed Death Ligands (PD-L1/2) and OX40 ligand (OX40L) [10,11,12,13]. DCs acquire immune regulatory functions by interaction with pathogens, including helminths, via various pattern-recognition receptors, such as toll-like receptors (TLRs) and C-type lectin receptors (CLRs) [14].

In this study we focus on defining molecular mechanisms that contribute to the suppression of inflammatory responses of human DCs by *S*. *mansoni* soluble egg antigens (SEA). Several studies have shown that production of pro-inflammatory cytokines by TLR-stimulated human DCs is markedly reduced in response to direct contact with SEA [15,16,17]. We have shown previously that SEA-primed human DCs induce a Th2 response *in vitro*, associated with increased expression of OX40L on DCs in the presence of LPS [17,18]. Interestingly, Omega-1, a major component of SEA, can condition DCs into an anti-inflammatory phenotype with potential to induce a Th2 response, which is proposed to occur mainly via its RNase action [19,20]. Other compounds within SEA include IPSE/α-1, which has been shown to induce IL-4 production [21] and Kappa-5 [22], however many compounds are still unidentified. DCs stimulated with miracidial fluid secreted by *S*. *mansoni* inhibit human T lymphocyte proliferation in mixed lymphocyte reactions (MLR) [23], indicating a reduction of the strength of the immune response. The underlying mechanism(s) by which SEA modulates human DCs, however, are still incompletely understood and likely include multiple SEA components and pathways.

*In vivo* mouse models have shown that the glycosylation of SEA is essential for its ability to induce anti-inflammatory Th2 responses [24,25]. SEA is recognized and internalized by human DCs via several CLRs, including DC-specific ICAM-3-grabbing non-integrin (DC-SIGN), the mannose receptor (MR) and macrophage galactose-type lectin (MGL), important receptors for internalization of glycosylated antigens and intracellular signaling upon binding of specific glycans [17,26,27,28]. Some major components within SEA have been identified that bind these CLRs. Omega-1 and IPSEα1, which both contain Galβ1-4(Fucα1–3)GlcNAc (Lewis X, Le^X^) motifs on diantennary N-glycans [29,30], interact with DCs mainly via the MR, whereas Kappa-5 interacts with all three CLRs [31]. It has been shown that after internalization of Omega-1 in the DCs by the MR, its RNase activity contributes to an anti-inflammatory DC phenotype by reducing protein synthesis via RNA breakdown [32]. Recent data show that SEA contains components that suppress TLR-induced proinflammatory cytokine expression via DC-SIGN by activation of NF-κB family member Bcl3 [33]. Thus, multiple components and mechanisms seem to play a role in immune modulation of DCs by SEA.

Using total SEA we here provide novel mechanistic evidence that SEA glycans contribute to the induction of an anti-inflammatory phenotype in human DCs.

## Materials and Methods

### Reagents

Human IL-4 and recombinant human GM-CSF were purchased from Immunotools (Germany). *Escherichia coli* LPS (strain 0111:B4) was purchased from Sigma-Aldrich (USA). For flow cytometric analysis PE-conjugated mouse anti-human OX40L (Ik-1,BD Pharmingen, 1:50) and anti-human PD-L1 (eBioscience, MIH1, 1:50) were used. Monoclonal anti-human DC-SIGN antibody was purchased from R&D systems (USA). In addition, anti-human DC-SIGN antibody AZN-D1 was used [34]. Anti-human CD206 (MMR) antibody was purchased from BD Pharmingen (USA, clone 19.2). IgG2b and IgG1κ control antibodies, for anti-DC-SIGN and anti-MR antibodies respectively, were purchased from BD (USA). Methyl α-D-mannoside (MeMan) was obtained from Sigma-Aldrich (USA). Allyl α-D-mannopyranoside (AllMan) was synthesized by standard Fischer glycosylation of allyl alcohol using D-mannose and acetyl chloride as acidic catalyst as described [35]. Purity of the product was confirmed by elemental analysis.

### Monocyte isolation and generation of human immature dendritic cells

Human monocytes were isolated from buffy coats of healthy donors (commercially obtained from Sanquin, Amsterdam, the Netherlands) or fresh blood of healthy volunteers, who gave written informed consent. Blood samples were collected from volunteers by a qualified technician. The authors had no interactions with the volunteers, and have no access to identifying information regarding the volunteers. The Medical Research Involving Human Subjects Act (WMO) does not apply to this study and an official approval of this study by the Medical Ethics Review Committee of VU University Medical Center is not required. The Medical Ethics Review Committee of VU University Medical Center is registered with the US Office for Human Research Protections (OHRP) as IRB00002991. The FWA number assigned to VU University Medical Center is FWA00017598.

Monocytes were isolated from peripheral blood mononuclear cells using CD14 microbeads (MACS, Miltenyi Biotec, Germany), as previously described [36] and differentiated into immature DCs in the presence of 250 IU/ml IL-4 and 120 IU/ml GM-CSF. For all experiments, monocyte derived DCs were used after 4 days of differentiation. In all DC assays LPS was used at a final concentration of 10 ng/ml. *S*. *mansoni* SEA was used at a final concentration of 40 μg/ml unless indicated otherwise. For all SEA and LPS co-incubations, SEA was added 15 minutes prior to LPS. In CLR blocking experiments, blocking compounds and antibodies were added to the DCs for 2 hours, followed by 4 hours of incubation with SEA and/or LPS.

### Denaturation and periodate treatment of *S*. *mansoni* SEA

Crude *S*. *mansoni* SEA was kindly made available by Dr. Fred Lewis (Biomedical Research Institute, Rockville, MD, USA), and purified as described previously [37]. To oxidize glycan moieties, SEA was treated with sodium periodate (PI) as previously described [38]. SEA was denatured (DN) by incubation for 20 min. at 80°C, then immediately cooled on ice and stored at -20°C until use. Endotoxin contamination was determined in all samples by Limulus Amoebocyte Lysate assay (Charles River Laboratories, Leiden, NL). Contamination was lower than 1 ng/ml endotoxin for all preparations. The SEA preparation used does not contain detectable levels of LPS in functional assays in DCs and TLR-reporter cells, tested as described [17].

### ELISA and DC-adhesion using immobilized SEA

SEA was immobilized in wells of Nunc Maxisorp plates (Thermo Scientific) in all experiments. Binding of the human recombinant hybrid CLRs DC-SIGN-Fc (2.5 μg/ml) [39], MGL-Fc (0.5 μg/ml) [28] and murine MR-Fc, containing the carbohydrate recognition domains CTLD4-7 (1 μg/ml, a kind gift from L. Martinez-Pomares) to SEA (5 μg/ml), was detected by peroxidase-conjugated anti-human Fc antibody (Jackson ImmunoResearch Laboratories, West Grove, PA, USA). Adhesion of (fluorescently-labelled) DCs to SEA (5–20 μg/ml as indicated), was performed essentially as previously described [40]. Fluorescence was measured at 485/520 nm with a FLUOstar spectrofluorometer (BMG Labtech, Ortenberg, Germany).

### Measurement of RNase activity

RNA of immature DCs was isolated using the RNeasy Mini Kit (Qiagen, Hilden, Germany) according to the manufacturer’s protocol. Untreated, DN- or PI-treated SEA (10 μg/ml) was added to 2 ng of RNA each and incubated at 37°C for 1 hour. The SEA-treated RNA samples and RNA control sample were loaded on an RNase free 2% agarose RNA gel and electrophoresis was carried out to separate the RNA molecules. Buffers and gels were autoclaved before use to prevent RNase contamination.

### Cytokine measurements

Culture supernatants were harvested after DC stimulation at indicated time points and frozen at -20°C until further analysis. TNFα was routinely measured by ELISA using the CytoSet ELISA kit (Biosource) according to the manufacturer’s protocol. For the multiplex bead immunoassay the Human Inflammatory 5-plex panel (Invitrogen, USA) was supplemented with a single-plex kit for IL-12 p70 (Invitrogen, USA). The assay was performed according to Invitrogen’s user manual ‘Human Inflammatory 5-Plex Panel’. Samples were measured by Luminex 200 (Bio-Rad, USA) and data analysis was performed using Bio-plex Manager 6.0 software. The TGF-β1 ELISA (Promega, G7591) was done according to the manufacturer’s protocol.

### Flow cytometry

For the determination of PD-L1 expression, buffycoat-derived DCs were stimulated with SEA for 24h. To measure OX40L, DCs were differentiated from fresh blood-isolated monocytes as described. Expression of OX40L on the cell surface was determined after a 48h co-incubation of the DCs with SEA antigens and LPS. Cell staining was analysed by flow cytometry using a BD FACSScan (Becton Dickson, USA). Data were analyzed using CellQuest Pro software (version 3.3, Becton Dickinson, USA).

### Mixed Lymphocyte Reaction

Immature DCs were stimulated for 24 hours. Subsequently, naïve CD4^+^ T cells were isolated using a negative selection naïve CD4^+^ T cell isolation kit (Miltenyi Biotec, Germany). DCs were counted and cocultured with immature CD4^+^ T cells for 6 days in triplicate for each donor. On day 5, tritium thymidine (0.02 mCi/ml) was added. Tritium thymidine incorporation was determined using the Unifilter-96 harvester (PerkinElmer, USA).

### Quantitative RT-PCR

DCs were harvested at time points indicated, and snap-frozen at -80°C. mRNA was isolated using the mRNA Capture kit (Roche, Switzerland), and subsequently transcribed into cDNA using the Reverse Transcription System kit (Promega, USA), as described previously [41]. PCR reactions were performed with the SYBR Green method as described previously [42]. Oligonucleotides were designed using Primer Express 2.0 (Applied Biosystems, USA) computer software. All oligonucleotides were synthesized by Ocimum (Ocimum Biosolutions, India). Primer sequences were designed as shown in Table 1.

**Table 1:** Oligonucleotide primers for quantitative RT-PCR.

Gene	Forward	Reverse
SOCS1	TGAACTCGCACCTCCTACCTCT	CAACCCCTGGTTTGTGCAA
SOCS2	TCTCTGCAGCATCTCTGTAGGC	GATGGCACCGGTACATTTGTT
SOCS3	TTTCTGATCCGCGACAGCT	TGACGCTGAGCGTGAAGAAGT
SHP1	ACTCCGTACCTTACAGGTCTCCC	GATCTCCCGAATCAGGTCTCC
SHP2	GAGAGAAAGGTGTTGACTGCGATA	CCTCTGAGACCGCACCATCT
PD-L1	ACAGCTGAATTGGTCATCCCA	TTCATTTGGAGGATGTGCCA
TGF-β	ACTATTGCTTCAGCTCCACGGA	AGTCAATGTACAGCTGCCGCA
GAPDH	CCATGTTCGTCATGGGTGTG	GGTGCTAAGCAGTTGGTGGTG

^SOCS, Suppressor of Cytokine Signalling; SHP, SH2-containing protein tyrosine Phosphatase; PD-L1, Programmed Death Ligand 1;TGF, Transforming Growth Factor; GAPDH, Glyceraldehyde 3-Phosphatase Dehydrogenase^

### SDS-PAGE and Western Blotting

DC samples were analyzed by SDS-PAGE and immunoblotting using rabbit-anti-human SOCS1 (Cell Signaling, 3950, 1:750), rabbit-anti-human SOCS2 (Cell Signaling, 2779, 1:500), goat-anti-rabbit-HRP (Dako, P0448 1:2000), mouse-anti-human GAPDH (9484 Abcam, UK, 1:1000), rabbit-anti-mouse-HRP (P0161, Dako, P0161, 1:2000), rabbit-anti-human SHP1 (GeneTex, 102865, 1:500) rabbit-anti-human SHP2 (Santa Cruz, C-18, 1:250) and goat-anti-human Actin (Santa Cruz, I-19, 1:2000). Proteins were detected using SuperSignal West Pico Chemiluminescent Substrate (Thermo Scientific, USA), in an EpiChemi II Darkroom (UPV Laboratory Products). Bands were quantified using Labworks 4.0 image acquisition and analysis software. DN or PI-treated and untreated SEA (10 μg/ml) were analysed by gel electrophoresis and silver staining according to the manufacturers protocol (Bio-Rad).

### Statistical analysis

Results are expressed as the mean plus or minus SEM. To correct for the large donor variation in cytokine production, the level of cytokine secretion induced upon LPS-stimulation was set at 100% for each donor. Statistical analyses were performed using SPSS 20.0 software (IBM, USA). For paired comparisons of two groups, a paired sample T-test was performed. For other parametric data, a one-way ANOVA was performed followed by Dunnett’s multiple comparisons test. * p<0.05; ** p<0.01, *** p<0.001.

## Results

### *S*. *mansoni* SEA glycans are involved in modulation of DC phenotype

**Fig 1:**
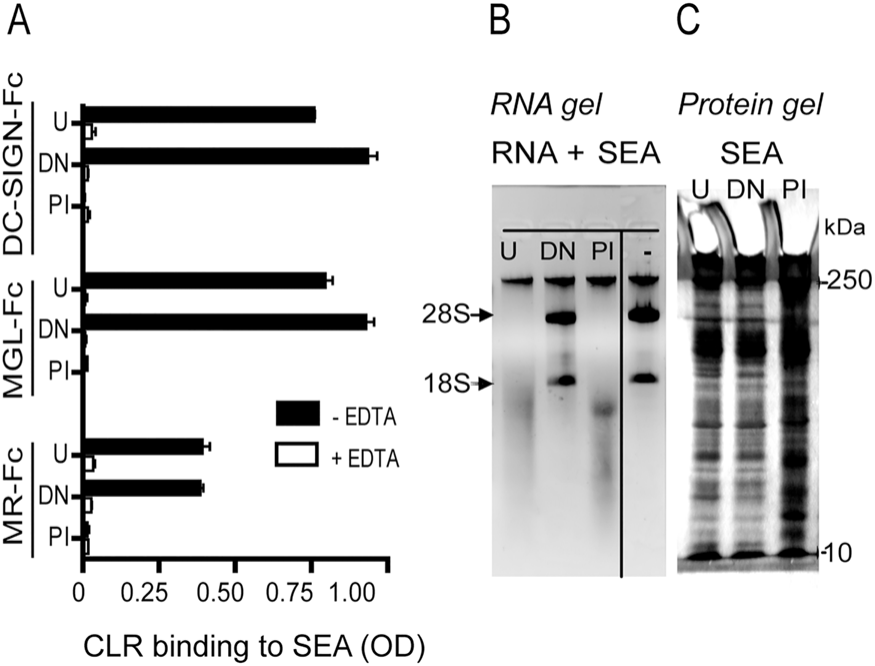
Treatments of *S*. *mansoni* SEA with periodate (PI) or heat (DN) results in modified glycan structures, or inactivation of RNase activity, respectively. **A**. Binding of IgG-Fc hybrid molecules of the C-type lectin receptors DC-SIGN, MGL and MR to untreated (U) SEA, SEA treated with 10 mM periodate (PI), or heated at 80°C for 20 min (DN) (coated at 5 μg/ml) was assessed by ELISA. In addition, binding to SEA was evaluated in the presence of EDTA (5 mM), which prevents CLR binding by removing calcium. **B**. RNase activity of the differentially treated SEA was assessed using total RNA isolated from human dendritic cells. All SEA samples were incubated with RNA for 1h at 37°C and subsequently agarose gel electrophoresis was performed under RNase-free conditions to visualize degradation of the RNA. The position of the 28S and 18S ribosomal units in the gel are indicated by arrows. **C**. Silver staining of an SDS-PAGE protein gel loaded with SEA U, DN and PI (10 μg/ml). The position of 10 and 250kD molecular weight markers on the gel are indicated by arrows. The data shown are typical examples out of 3 independent experiments.

*S*. *mansoni* SEA has previously been shown to modulate DC maturation, including the suppression of TLR-induced pro-inflammatory cytokine production, in a concentration dependent manner [17]. However, the mechanisms by which SEA acts on human DCs are incompletely understood. To discriminate between possible glycan- and RNase-mediated effects of the helminth antigens, SEA was either heat-denatured (DN) to abolish RNase activity, or periodate (PI) treated, to oxidize glycan structures [43]. To confirm the effectiveness of PI treatment, the binding capacity of different CLRs to the treated SEA preparations was measured. PI treatment caused loss of binding by the recombinant CLR-Fc hybrids MGL-Fc, DC-SIGN-Fc and MR-Fc to SEA (Fig 1A), indicating that the glycan ligands for these CLRs were destroyed by PI treatment. By contrast, heat treatment (DN) of SEA did not affect lectin binding, indicating the presence of intact glycan ligands on the heat-treated antigens. Heat treatment did, however, abolish the capacity of SEA to degrade RNA (Fig 1B), indicating that RNase activity was destroyed, whereas PI treatment did not destroy the RNase activity within SEA. The mild DN and PI treatments did not result in precipitation or loss of SEA proteins, as indicated by SDS-PAGE analysis of the final sample (Fig 1C).

**Fig 2:**
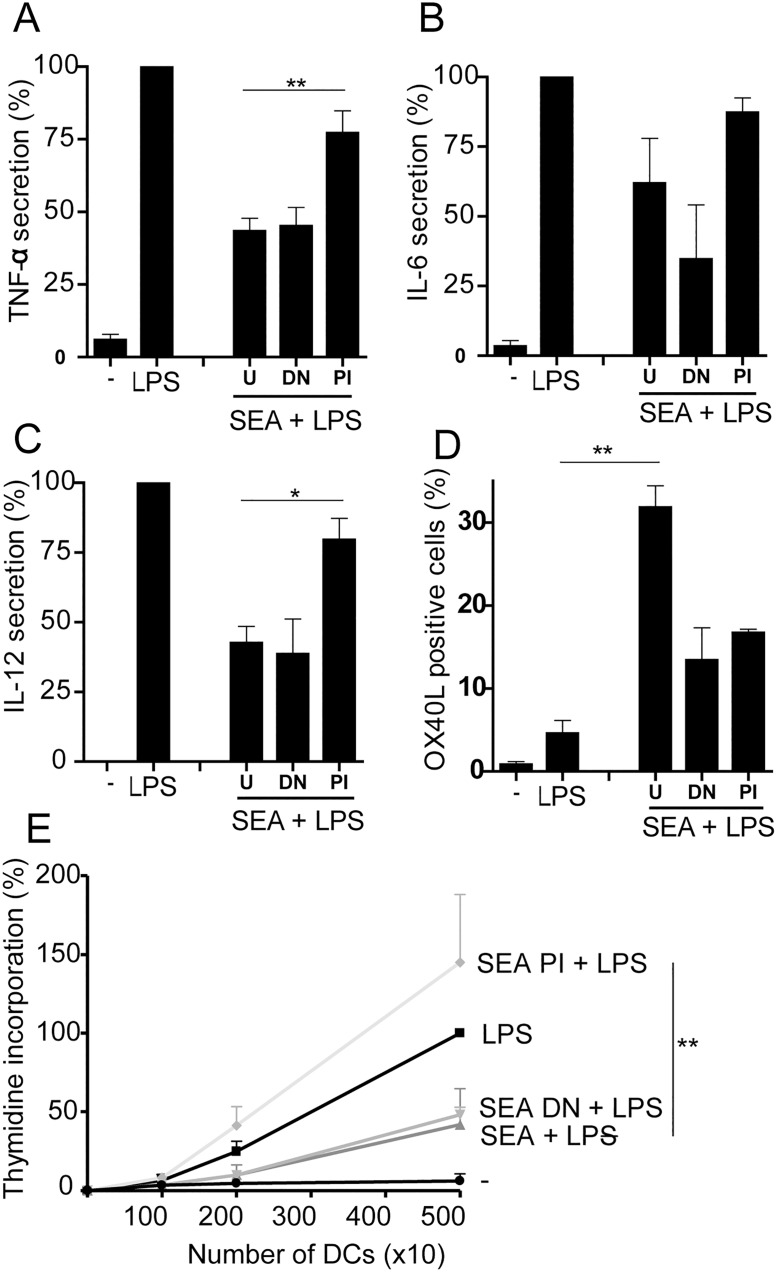
The ability of *S*. *mansoni* SEA to suppress LPS-induced human dendritic cell (DC) activation and OX40L expression on DCs is reduced after periodate treatment of SEA. DCs were incubated with LPS, with or without either untreated (U), heat treated (DN) or periodate (PI) treated SEA (40 μg/ml) for 24h. In **A**, **B**, and **C**, respectively is shown the secretion of TNFα, IL-6 and IL-12, which were determined in the supernatant by Luminex multiplex bead analysis or ELISA. Experiments using SEA alone did not affect cytokine production of DCs (controls, not shown). Cytokine secretion by LPS alone was set at 100% separately for each donor and varied for TNFα from 100–550 ng/ml, for IL-6 from 4–20 ng/ml and for IL-12 from 50–200 ng/ml, dependent on the blood donor used. **D**. The expression of OX40L on the DC surface was assessed by flow cytometry after 48h. **E**. A mixed lymphocyte reaction was done by priming the DCs for 24h with LPS in the presence or absence of SEA, followed by coculture of the primed DCs with heterologous naïve CD4^+^ T cells for 6 days. Tritiated thymidine incorporation was determined during the final 24h of incubation. Results are expressed as mean plus or minus SEM and reflect the mean of experiments performed in duplicate with DCs from 8 (A), 4 (B, C, E) or 3 (D) separate donors. Significance is *p<0.05, **p<0.01 vs untreated (A-C,E) or LPS alone (D).

After establishing the efficacy of both heat and periodate treatments, human DCs were pulsed with treated and untreated SEA in the presence of LPS, and their immunomodulatory capacities were analysed. The results in Fig 2 establish that secretion of the LPS-induced cytokines tumor necrosis factor α (TNFα), IL-6 and IL-12p70 by DCs was suppressed by SEA and show that this suppression was not significantly affected by heat-treatment of the antigens, indicating that RNase activity does not significantly contribute to the observed effects. In contrast, PI treatment of SEA did abolish its ability to suppress these cytokines (Fig 2A–2C). In addition, CD4^+^ T cell proliferation, as measured by an MLR, was suppressed by the addition of either untreated or heat-treated SEA (Fig 2E), but not by PI-treated SEA. The enhanced OX40L expression induced by SEA in the presence of LPS was inhibited by either heat-treatment or PI-treatment (Fig 2D), indicating the importance of both an intact glycan and protein structure here. These data indicate that SEA-glycans significantly contribute to the modulation of DC function.

### *S*. *mansoni* SEA induces expression of different immunomodulators in human DCs

**Fig 3:**
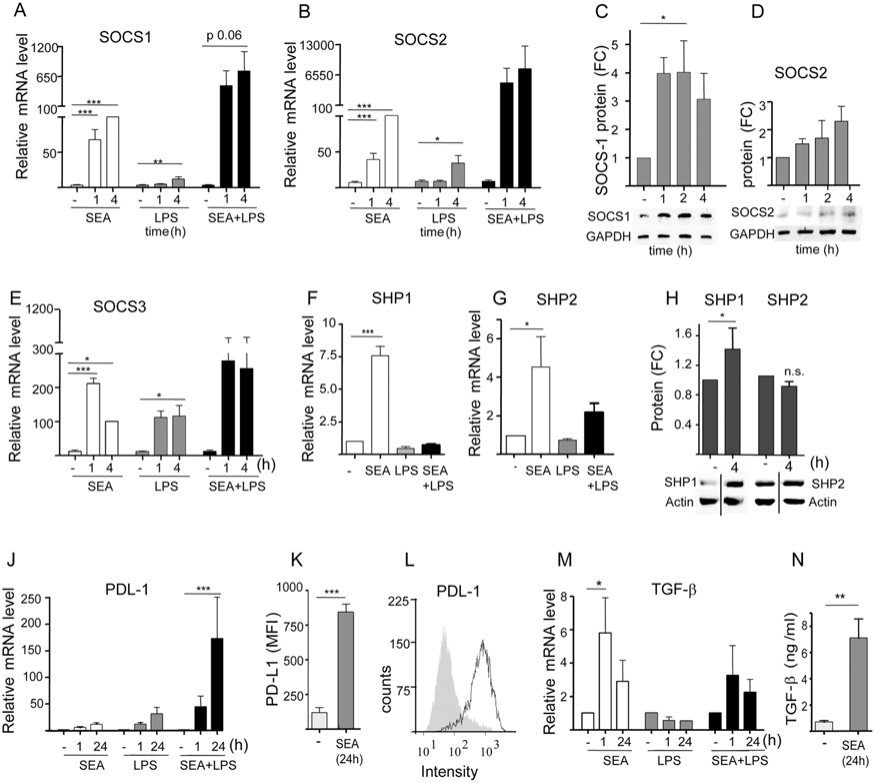
*S*. *mansoni* SEA induces mRNA and protein expression levels of Suppressor of Cytokine Signaling 1 (SOCS1), SH2-containing protein tyrosine phosphatase 1 (SHP1), Programmed Death Ligand 1 (PD-L1) and transforming growth factor β (TGF-β), in human dendritic cells (DCs). DCs were cultured with SEA (40 μg/ml) and/or LPS (10 ng/ml) for the indicated time periods. mRNA levels were detected using quantitative real time PCR with GAPDH as a reference gene, whereas protein levels were determined by SDS-PAGE and western blotting, using specific antibodies and reference gene antibodies on the same blots. Quantification of protein was performed by density scanning. In **A**, **B**, and **E**, respectively, the mRNA levels of SEA-induced SOCS1, SOCS2, and SOCS3 are shown, whereas the protein levels of SOCS1 and SOCS2 are shown in **C** and **D**, respectively. In **F** and G, respectively, the mRNA levels of SEA-induced SHP1 and SHP2 are shown, and their protein levels are shown in **H**. The mRNA levels of PDL-1 and TGF-β are shown in **J** and **M**, respectively. Protein levels of PD-L1 were determined by flow cytometry (**K)** and TGF-β1 protein levels were determined by ELISA (**N**). **L** shows a typical example of a FACS plot from one of the experiments shown in **K**. Results are shown as mean fold difference from unstimulated DCs, plus or minus SEM. Results are derived from experiments performed in duplicate using DCs derived from 7 (A), 4 (B, D, E, G, H, J, N) or 3 (C, F, M, K) different donors. Significance is indicated versus unstimulated DCs as *p<0.05, **p<0.01 and ***p<0.001.

To provide insights into the pathways used by SEA for human DC modulation, mRNA levels of known negative regulators of the TLR4 signalling pathway were determined [44,45]. DCs were incubated with SEA in the presence and absence of LPS. Our data show that SEA significantly induced mRNA levels of SOCS1 either alone or in combination with LPS, with a further enhanced response upon costimulation with LPS (Fig 3A). In addition, SEA induced SOCS2 mRNA levels (Fig 3B) and SOCS3 levels (Fig 3E). It has previously been shown that SEA-stimulated DCs induce SOCS3 protein levels [46]. Determination of SOCS1 and SOCS2 protein levels by western blotting showed that SOCS1 levels were increased at 1h after DC stimulation with SEA and remained increased for several hours (Fig 3C), whereas induction of SOCS2 protein levels was not significant (Fig 3D). SEA treatment of DCs also enhanced the expression of SHP1 and SHP2 mRNA levels (Fig 3F and 3G). Determination of SEA-induced SHP protein levels showed that although the protein levels of SHP2 remained unaltered upon DC stimulation with SEA, expression of SHP1 protein was enhanced after 4h (Fig 3H).

Furthermore, DC stimulation with SEA resulted in the induced expression of the surface marker PD-L1 and the cytokine TGF-β both on the mRNA level (Fig 3J and 3M) and the protein level (Fig 3K, 3L and 3N).

### SOCS1 and SHP1 are not induced upon contact of DCs with immobilized *S*. *mansoni* SEA

**Fig 4:**
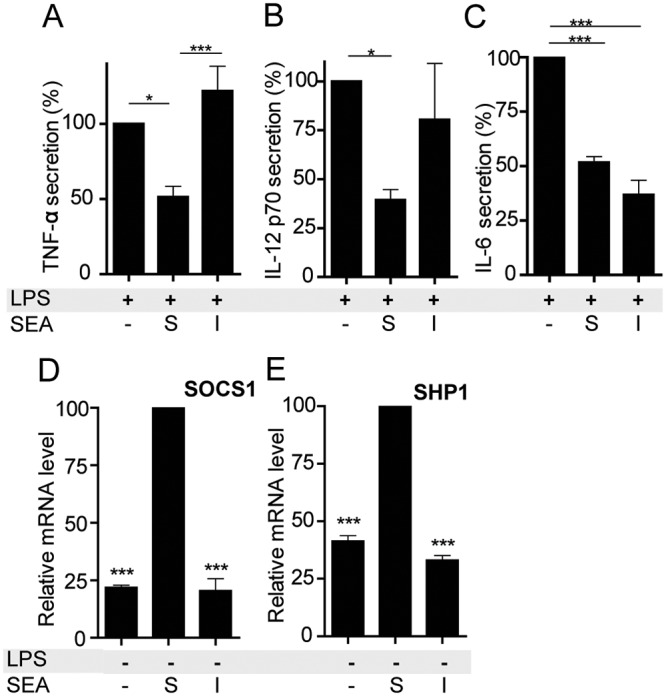
The ability of *S*. *mansoni* SEA to induce SOCS1 and SHP1 expression and to suppress TNFα production in human dendritic cells (DCs) is lost by immobilization of SEA. DCs were incubated with 20 μg/ml soluble SEA (S) or immobilized SEA (I) with or without LPS. SEA was coated to wells of an ELISA plate, and the plate was washed before DCs were added, **A-C** The production of the cytokines TNFα (**A**), IL-12p70 (**B**) and IL-6 (**C**) was determined in the DC supernatants after 4h (IL-6 and TNFα) or 24h (IL-12p70). The cytokine production of LPS-activated DCs was set at 100% in each experiment and varied for TNFα from 40–200 ng/ml, for IL-6 from 2–10 ng/ml and for IL-12 from 50–200 ng/ml, dependent on the blood donor used. **D-E** Relative mRNA levels of SOCS1 (**D**) and SHP1 (**E**) were determined in the cells after 4h by quantitative real-time PCR using GAPDH as reference gene. Results are shown from experiments performed in duplicate using DCs derived from 5 (A), 4 (B, C) or 3 (D, E) different blood donors, and are displayed as the mean plus or minus SEM. Significance is indicated versus LPS-stimulated DCs (**A-C**) or SEA-stimulated DCs (**D-E**) as *p<0.05, **p<0.01 and ***p,0.001.

In regard to the mechanism by which SEA negatively regulates TLR4 signaling, we hypothesized that *S*. *mansoni* SEA has to be recognized and internalized by lectin-receptors present on DCs to suppress the cytokine production from LPS-activated DCs, and/or to enhance expression levels of SOCS1 and SHP1. To test this possibility, SEA was presented to DCs either soluble in the culture medium, or immobilized to a plate to prevent internalization. Immobilization of SEA resulted in loss of its capacity to suppress the LPS-induced production of TNFα and possibly IL-12p70 in DCs, whereas suppression of IL-6 production remained unaltered (Fig 4A–4C). In addition, immobilization caused a complete loss of the expression of both SOCS1 and SHP1 (Fig 4D and 4E). These data show that a complete loss of induction of both SOCS1 and SHP1 expression is associated with a loss of suppression of TNFα, but not IL-6 production, indicating a difference between the regulation of expression or secretion of these cytokines. In addition, these data suggest that uptake of SEA by DCs is required for their ability to suppress LPS-induced TNFα production and to enhance SOCS1 and SHP1 expression.

### *S*. *mansoni* SEA-induced SOCS1 and SHP1 expression is reduced by blocking its interaction with the MR

**Fig 5:**
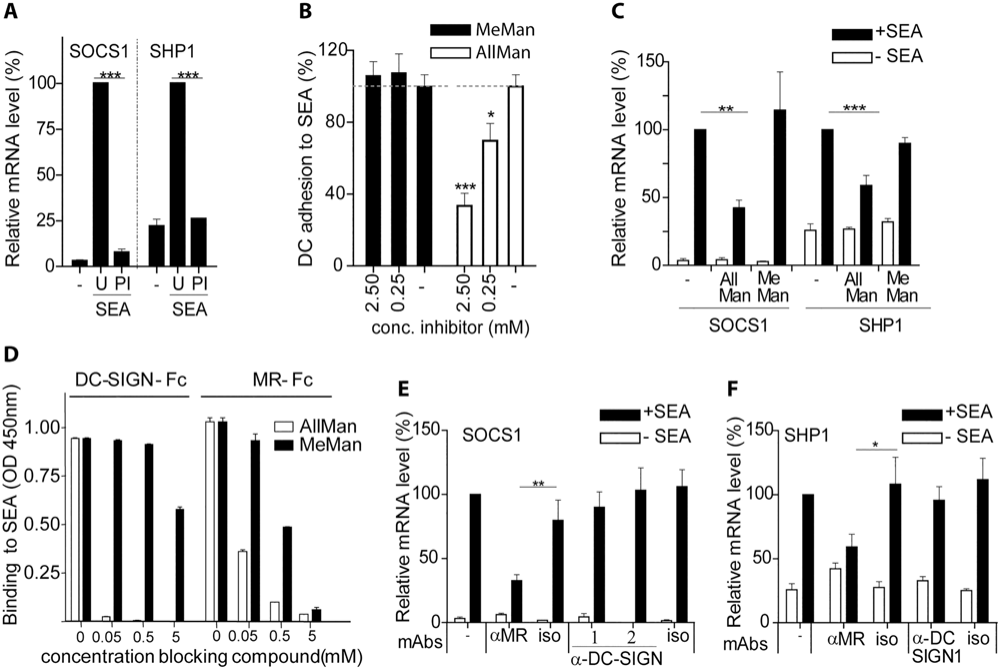
*S*. *mansoni* SEA induces SOCS1 and SHP1 in human dendritic cells (DCs) via the mannose receptor (MR). **A**. DCs were incubated with untreated (U) or periodate (PI) treated SEA (40 μg/ml). After 4h, SOCS1 and SHP1 mRNA levels were determined by quantitative real-time PCR using GAPDH as a reference gene. **B**. Binding of DCs to SEA (coated at 5 μg/ml) was determined in the presence or absence of different concentrations of the mannose derivates AllMan or MeMan. Binding of the DCs to SEA was set at 100% for each donor. **C**. DCs were pre-incubated for 2h with 2.5 mM of AllMan or MeMan, followed by incubation with SEA (40 μg/ml) for 4h. Relative mRNA levels of SOCS1 and SHP1 were determined by quantitative real time PCR using GAPDH as a reference gene. **D**. Inhibition of DC-SIGN-Fc and MR-Fc binding to SEA (coated at 5 μg/ml) was detected in the presence of low concentrations of AllMan (0.5–5 mM). **E-F**. DCs were pre-incubated for 2h with anti-MR, anti-DC-SIGN1 or anti-DC-SIGN2 mAbs (5 μg/ml), or their respective isotype control mAbs, followed by incubation with SEA (40 μg/ml) for 4h. Relative mRNA levels of SOCS1 (**E**) and SHP1 (**F**) were determined by quantitative real time PCR using GAPDH as a reference gene. All data shown are derived from experiments performed in duplicate using DCs of 4 (A, C), 3 (B) or 5 (E, F) different donors. Results are shown as the mean plus or minus SEM. In **A**, **C**, **E** and **F**, DCs incubated with untreated SEA were set at 100%. Significance is indicated compared to DCs stimulated with untreated SEA as *p<0.05, **p<0.01 and ***p<0.001.

SEA has previously been shown to be internalized via the glycan binding CLRs DC-SIGN, MGL and MR [17]. Thus, we hypothesized that induction of SOCS1 and/or SHP1 may depend on internalization of SEA by DCs via interaction of SEA-associated glycans with one or more of these CLRs. To evaluate this possibility, SOCS1 and SHP1 mRNA levels were determined in DCs pulsed with either PI-treated or untreated SEA. Interestingly, PI treatment of SEA resulted in a reduced mRNA expression of SOCS1 and SHP1 (Fig 5A), indicating that SEA glycans contribute to the enhanced expression of both genes. To further establish a role for SEA glycans in SOCS1 and SHP1 expression, we investigated glycans that could inhibit the binding of DCs to SEA, thereby possibly also inhibiting SEA-induced SOCS1 and/or SHP1 expression. Since DC survival was affected by using high concentrations of free monosaccharides, necessary to enable blocking of DC binding to SEA, we tested a panel of potentially more potent monosaccharide derivatives for their ability to inhibit binding of DCs to SEA. We found that the mannose-derivative allyl α-d-mannoside (AllMan) inhibited the binding of DCs to SEA at low concentrations, in contrast to methyl α-D-mannoside (MeMan) (Fig 5B). Interestingly, AllMan also inhibited SEA-induced SOCS1 and SHP1 expression by DCs, whereas MeMan did not show inhibition at a similar concentration (Fig 5C). Incubation of DCs with AllMan or MeMan alone did not alter expression levels of SOCS1 or SHP1. These results establish that specific SEA-associated glycans are essential for the induced SOCS1 and SHP1 expression in DCs, possibly via interaction with a CLR with mannose-specificity. To further test the specific involvement of CLRs, we then tested whether AllMan could block the binding of DC-SIGN-Fc and/or MR-Fc, both mannose/fucose recognizing CLRs [47,48], to SEA. We found that low concentrations of AllMan blocked binding of both MR-Fc and DC-SIGN-Fc to SEA (Fig 5D), whereas higher MeMan concentrations were needed to achieve a partial suppression of MR-Fc and DC-SIGN-Fc interaction with SEA.

To discriminate between the putative involvement of DC-SIGN and the MR in the SEA-induced expression of SOCS1 and SHP1, DCs were incubated with blocking antibodies for the MR or DC-SIGN prior to SEA stimulation. The data indicate that the blocking antibodies to MR, but not isotype control antibodies, significantly inhibit SEA-induced SOCS1 and SHP1 expression. By contrast, blocking of DC-SIGN did not affect SOCS1 and SHP1 expression (Fig 5E and 5F). In addition, we determined whether blocking the MR or DC-SIGN using similar concentrations and conditions as described above, could prevent the potential of SEA to suppress LPS-induced production of pro-inflammatory cytokines. However, we were unable to find a significant effect (data not shown), suggesting that a more complete block of the MR, or a contribution of other yet unknown factors, is required for an effective suppression of cytokine production. In summary, these data indicate that SEA glycans are essential to induce expression of SOCS1 and SHP1 in human DCs via interaction with the MR.

## Discussion

Several studies by our group and others have shown that production of pro-inflammatory cytokines by DCs is markedly reduced in response to direct contact with helminth antigens [17,18,49]. In mouse models it was shown that oxidation of the glycans of SEA, which contains many glycoproteins, affected the capacity of SEA to induce a Th2 response, indicating the involvement of SEA glycans in this process [24,25]. We here set out to determine the role of glycan determinants of SEA in the modulation of LPS-induced activation of human DCs and subsequent T cell proliferation. Recently it was shown that Omega-1, a major glycoprotein in SEA, can polarize DCs into a Th2 promoting phenotype, which was attributed to both the glycosylation and RNase action of Omega-1 [20,32]. To discriminate between putative glycan-mediated effects of SEA and effects associated with RNase activity, we subjected SEA to either mild periodate treatment to oxidize glycan structures and thus destroy their integrity, or heated the SEA for 20 min at 80°C to denature protein structures thereby abolishing RNase activity. Our data revealed that PI-sensitive SEA glycans regulate important properties of human DC function. Such glycans contribute to the suppression of the LPS-induced secretion of TNFα, IL-6 and IL-12 by DCs, whereas heat-treatment of these products abolished RNase activity, but did not affect the observed suppressive properties of SEA. Importantly, we show that PI treatment of SEA, but not heat-treatment, reduced the capacity of SEA-primed DCs to induce T cell proliferation. The data presented here show that RNase activity is not a dominant factor within total SEA in its contribution to the described anti-inflammatory properties in DCs, whereas periodate sensitive glycans of SEA do play a significant role.

The novel finding that SEA increases the expression levels of SOCS1 and SHP1, known negative regulators of TLR4 signalling, provides insight into the mechanisms by which SEA suppresses TLR4-induced DC activation [45]. SOCS1 belongs to a family of proteins that inhibits the cytoplasmic JAK/STAT signalling pathway induced in TLR signalling, and has been shown to be of crucial importance for the limitation of inflammatory responses [44,50,51]. SHP1 is part of the protein tyrosine phosphatase family and has been reported to play a role in the negative regulation of TLR-mediated immune responses, including those induced via TLR4 [45]. Remarkably, deficient macrophage SHP-1 expression has been observed in MS patients, and is correlated with an enhanced inflammatory phenotype [52]. The induction of negative regulators of TLR signalling may be a strategy of pathogens to limit pro-inflammatory responses. Recently, elevated SOCS3 levels were observed in SEA-treated DCs [46], and elevated SOCS1 expression was observed in HIV- and *Mycobacterium avium*-infected patients [53,54].

Our data show that SEA strongly increases SOCS1 mRNA levels in human DCs. Whereas posttranscriptional regulation plays an important role in maintaining the stability of SOCS1 protein [55,56], our data show enhanced SOCS1 protein levels in DCs at 1h after addition of SEA, and these levels are maintained for several hours. This indicates that SEA induces significant levels of stably expressed SOCS1 protein. In addition, SHP1 mRNA and protein levels are induced by stimulation of DCs with SEA under similar conditions as SOCS1 activation. Our data also reveal that the capacity of SEA to induce SOCS1 and SHP1 is glycan-dependent, since both oxidation of SEA glycans and blocking the binding of SEA to DCs by adding the mannose-mimic AllMan inhibit the induction of SOCS1 and SHP1 expression. Remarkably, SEA induces a simultaneous upregulation of SOCS1 and SHP1 in all assays, indicating that co-regulation of both negative regulators may occur. It has been demonstrated that SOCS1 can contribute to SHP1 function in the negative regulation of cytokine signalling, and that loss of SOCS1 can significantly compromise the ability of SHP1 to down-regulate STAT5 activation [57].

The involvement of SEA glycans in the modulation of DC function implicates a role for one or more lectin receptors on DCs. CLRs are known to internalize soluble glycoconjugates, and are able to induce intracellular signaling cascades, thereby enhancing or suppressing TLR signal transduction routes [58,59]. We have shown previously that DCs internalize SEA via the CLRs DC-SIGN, MGL and the MR [17]. Here, we demonstrate that SOCS1 and SHP1 induction can be blocked both by the mannose derivative AllMan and by anti-MR antibodies indicating that the SEA-induced expression of SOCS1 and SHP1 in DCs is MR-mediated.

The MR is an endocytic receptor and cross-linking of the MR can enable the induction of an immunosuppressive phenotype in DCs [60], although no intracellular signaling motif has been found in its cytoplasmic tail [61]. The MR has previously been shown to play a significant role in the *S*. *mansoni*-induced modulation of pro-inflammatory responses in murine macrophages via internalization of excretory/secretory material released by cercariae [62]. The mechanism by which the MR induces SOCS1 and SHP1 expression is yet to be determined. The capacity of SEA to upregulate mRNA levels of SOCS1 and SHP1 via the MR is difficult to associate with RNase activity of Omega-1. It would be possible though that Omega-1 or Ipse/α-1, which show similarity in their glycosylation and are both internalized via the MR, could induce the upregulation of these negative regulators via their glycans, but it should be noted that more glycoproteins are present within SEA that, based on their glycomic profile [29,31,63,64], could be candidates for recognition and internalization by the MR. Although blocking SOCS1 and SHP1 expression with anti-MR Ab or AllMan resulted in a significant reduction of SOCS1 (~60%) and SHP1 (~40%) expression, we did not observe a significant reduction in the suppression of TNFα production of LPS-activated DCs. Possibly, the MR blockade by antibody is not sufficiently efficient to obtain an effect on the level of cytokine secretion, since a complete block of both SOCS1 and SHP1 expression by immobilization of SEA did reverse the suppression of TLR4-induced TNFα production. Alternatively, internalization of SEA via multiple receptors may be required to act in concerted action to induce expression of SOCS1 and SHP1 effectively. In addition, it is possible that multiple compounds of SEA can down-regulate the TLR4-induced cytokine production via different pathways, thereby acting as back-up systems for each other.

In summary, our results show that periodate sensitive glycans of *S*. *mansoni* SEA are essential for suppression of TLR4-induced pro-inflammatory responses in human DCs. SEA induces many phenotypic changes in DCs which are associated with anti-inflammatory properties, whereas RNase activity within SEA is not a dominant factor to condition DCs to suppress TLR4-induced cytokine production and T cell proliferation. In addition, our data provide novel insights in the mechanisms by which SEA glycans act and implicate a crucial role of the MR in the SEA-induced upregulation of SOCS1 and SHP1 expression in DCs. This may contribute to the capacity of SEA to down-regulate inflammatory responses in its host by modulation of adaptive T cell responses.
